# Clinical and radiological correlations in patients with gestational trophoblastic disease*

**DOI:** 10.1590/0100-3984.2015.0073

**Published:** 2016

**Authors:** Lana de Lourdes Aguiar Lima, Raphael Câmara Medeiros Parente, Izildinha Maestá, Joffre Amim Junior, Jorge Fonte de Rezende Filho, Carlos Antonio Barbosa Montenegro, Antônio Braga

**Affiliations:** 1 Master's Student in the Perinatal Health Program at the Faculdade de Medicina da Universidade Federal do Rio de Janeiro (UFRJ), Rio de Janeiro, RJ, Brazil.; 2 PhD, Physician at the Gynecology Institute of the Universidade Federal do Rio de Janeiro (UFRJ), Rio de Janeiro, RJ, Brazil.; 3 PhD, Adjunct Professor of Obstetrics at the Faculdade de Medicina da Universidade Estadual Paulista "Júlio de Mesquita Filho" (Unesp), Botucatu, SP, Brazil.; 4 PhD, Associate Professor of Obstetrics at the Faculdade de Medicina da Universidade Federal do Rio de Janeiro (UFRJ), Director of the Maternidade Escola da Universidade Federal do Rio de Janeiro (UFRJ), Rio de Janeiro, RJ, Brazil.; 5 PhD, Full Professor of Obstetrics at the Faculdade de Medicina da Universidade Federal do Rio de Janeiro (UFRJ), Rio de Janeiro, RJ, Brazil.; 6 PhD, Full Member Emeritus of the Academia Nacional de Medicina, Full Professor of Obstetrics at the Faculdade de Medicina da Universidade Federal do Rio de Janeiro (UFRJ), Rio de Janeiro, RJ, Brazil.; 7 PhD, Adjunct Professor of Obstetrics at the Faculdade de Medicina da Universidade Federal do Rio de Janeiro (UFRJ), Rio de Janeiro, RJ, and at the Faculdade de Medicina da Universidade Federal Fluminense (UFF), Niterói, RJ, Brazil.

**Keywords:** Hydatidiform mole, Gestational trophoblastic disease, Radiology

## Abstract

Gestational trophoblastic disease is an abnormality of pregnancy that encompasses a group of diseases that differ from each other in their propensity for regression, invasion, metastasis, and recurrence. In the past, it was common for patients with molar pregnancy to present with marked symptoms: copious bleeding; theca lutein cysts; uterus larger than appropriate for gestational age; early preeclampsia; hyperemesis gravidarum; and hyperthyroidism. Currently, with early diagnosis made by ultrasound, most patients are diagnosed while the disease is still in the asymptomatic phase. In cases of progression to trophoblastic neoplasia, staging-typically with Doppler flow studies of the pelvis and chest X-ray, although occasionally with computed tomography or magnetic resonance imaging-is critical to the choice of an appropriate antineoplastic therapy regimen. Because it is an unusual and serious disease that affects women of reproductive age, as well as because its appropriate treatment results in high cure rates, it is crucial that radiologists be familiar with gestational trophoblastic disease, in order to facilitate its early diagnosis and to ensure appropriate follow-up imaging.

## Abstract (pt)

Doença trofoblástica gestacional é anomalia da gravidez que engloba um grupo de doenças derivadas do trofoblasto, diferentes entre si na propensão para regressão, invasão, metástase e recidiva. No passado, era comum a paciente portadora de gravidez molar apresentar sintomas exuberantes: hemorragia copiosa, cistos tecaluteínicos, útero aumentado para a idade gestacional, pré-eclâmpsia precoce, hiperêmese e hipertireoidismo. Atualmente, com o diagnóstico precoce feito pela ultrassonografia, a maioria das pacientes é diagnosticada ainda na fase assintomática. Nos casos em que há progressão para neoplasia trofoblástica gestacional, o estadiamento feito com a dopplerfluxometria pélvica e a radiografia de tórax, eventualmente com a tomografia computadorizada e a ressonância magnética, é fundamental na avaliação da escolha do tratamento antineoplásico. Por ser uma doença incomum e grave, que acomete mulheres no menacme, mas cujo tratamento adequado determina elevadas taxas de cura, é fundamental que os radiologistas estejam familiarizados com essa entidade clínica, a fim de auxiliar no diagnóstico precoce e promover seu correto acompanhamento imaginológico.

## INTRODUCTION

Gestational trophoblastic disease (GTD) comprises a group of disorders arising from the anomalous growth of trophoblastic tissue. It presents a benign clinical spectrum represented by hydatidiform mole, either partial or complete, and by gestational trophoblastic neoplasia (GTN)-invasive mole, choriocarcinoma, placental site trophoblastic tumor (PSTT), and epithelioid trophoblastic tumor (ETT) characterizing the malignant forms^(1-4)^.

In most cases of GTN, cure can be achieved^(4)^. To that end, the radiologist should play an important role in its early diagnosis, decreasing the morbidity and mortality of molar pregnancy, as well as in the staging and follow-up of patients with GTN, guiding the rigorous and systematic treatment. The initial test used for the diagnosis of hydatidiform mole is ultrasound, in combination with determination of the level of the beta subunit of human chorionic gonadotropin (β-hCG) in serum^(1,3)^. Although chest X-ray is recommended as an initial means of screening for metastases, computed tomography (CT) and magnetic resonance imaging (MRI) have generally been incorporated into the evaluation of metastatic disease^(2,4)^, especially in more complex clinical cases^(2)^.

In this review, we present clinical and radiological correlations in patients with GTD, describing the diagnostic requirements for the use of the various ancillary methods, together with details regarding their radiological aspects and therapeutic utility in GTD, as well as summarily updating the information about this important complication of pregnancy.

## EPIDEMIOLOGY OF GTD

Accounting for 80% of all cases of GTD, hydatidiform mole reportedly occurs in 0.6-1.1/1000 pregnancies in North America^(5)^. It is estimated that hydatidiform mole occurs in 1/200-400 pregnancies in Brazil^(6)^, approximately 5-10 times more frequently than in North America and Europe^(7)^, perhaps reflecting differences in dietary or genetic influences.

As a cause of GTD, choriocarcinoma is rarer than is hydatidiform mole, the former occurring in 1/20,000-40,000 pregnancies ^(8)^. Currently, there are few data in the literature on PSTT and ETT. A recent study conducted in the United Kingdom suggested that PSTT and ETT collectively account for only 0.2% of cases of GTD^(9)^.

Women over 45 years of age are at an increased risk of molar pregnancy, as are those under 16 years of age, those with a history of molar pregnancy (a 1% increase after one such pregnancy and a 15-20% increase after two), and those with a history of miscarriage^(7)^.

### Etiopathogenesis of hydatidiform mole

Hydatidiform mole constitutes an error of fertilization. Complete hydatidiform mole originates from the fertilization of an oocyte without maternal chromosomes by a haploid sperm with subsequent duplication of paternal DNA, giving rise to an egg of exclusively parthenogenetic origin, with a diploid (46,XX) karyotype. In less than 10% of cases, complete hydatidiform mole arises when an oocyte without genetic material is fertilized by two separate sperm (dispermy), resulting in an egg of exclusively androgenetic origin, with a 46,XX or 46,XY karyotype. That aberration does not allow the formation of embryonic tissue or its attachments. Partial hydatidiform mole results from the fertilization of a normal egg by two sperm, resulting in a zygote with a triploid (69,XXY or 69,XXX) diandric karyotype^(10)^. In such cases, it is common to identify an embryo, or even a fetus, that is malformed and has anomalous attachments.

### Clinical aspects of hydatidiform mole

Patients with complete hydatidiform mole classically present with specific symptoms-vaginal bleeding (in 84%), increased uterine volume (in 50%), theca lutein cysts (in 40%), and high serum levels of β-hCG (in 50%)-whereas those with partial hydatidiform mole present with symptoms that mimic those of a common abortion^(11)^. Certain signs and symptoms of hydatidiform mole have become uncommon because the systematic use of ultrasound has resulted in the early (first-trimester) detection of pregnancy. Such signs and symptoms include anemia, hyperemesis gravidarum, hyperthyroidism, respiratory failure, and preeclampsia^(12)^.

### Diagnosis and treatment of hydatidiform mole

The diagnosis of hydatidiform mole should be based on a combination of clinical history, physical examination, serum β-hCG determination, and ultrasound^(1,13)^. The serum β-hCG level (typically > 100,000 mIU/mL) can vary greatly in normal and multiple pregnancies, as well as in molar pregnancy, and can therefore confound the diagnosis when considered in isolation^(3)^. Therefore, ultrasound is considered the principal method of diagnosing hydatidiform mole^(1,6,11)^.

After being diagnosed, patients with GTD should be evaluated at a referral center for its treatment, where the uterine contents can be evacuated by vacuum aspiration^(1,2)^. Because it has a lower risk of uterine perforation, vacuum aspiration is preferable to curettage. Histopathological examination should be performed in order to confirm the diagnosis and to identify the histological type of the hydatidiform mole.

The serum level of β-hCG is expected to decline after uterine evacuation, and subsequent measurements are performed weekly. After three normal results, serum β-hCG levels are determined on a monthly basis for the next six months, in order to detect recurrence and malignancy^(13)^. Patients should not attempt to conceive avoided during this period.

## GTN

According to the International Federation of Gynecology and Obstetrics (FIGO), GTN is identified by the following criteria^(14)^: a plateau in the serum β-hCG level lasting for more than three weeks (on days 1, 7, 14, and 21) an elevated serum β-hCG level for more than two weeks (days 1, 7, and 14); a histopathological diagnosis of choriocarcinoma; and an elevated serum β-hCG level for six months or more after uterine evacuation. The occurrence of GTN after complete hydatidiform mole ranges from 18% to 29%, compared with 0.5% to 11% after partial hydatidiform mole^(12)^.

The most common type of GTN is invasive mole, because, in most cases, the diagnosis is made when the cancer is still confined to the uterus^(15)^. Choriocarcinoma is a rarer type that often generates distant metastases. Characteristically, choriocarcinoma is associated with extensive tissue necrosis and hemorrhage^(16)^.

Less than 1% of cases of GTN are PSTT, which differs from the other types in that it produces low levels of β-hCG, has high human placental lactogen immunoreactivity, and features indolent cell growth, with a tendency to metastasize. Another extremely rare variant is ETT, which is having similar to PSTT in terms of its clinical behavior^(17)^.

After being diagnosed with GTN, patients should first be screened for metastases. We followed the guidelines established by the European Society of Medical Oncology, which recommend an initial assessment by Doppler flow study of the pelvis and chest X-ray. In patients showing lung metastases, brain MRI and abdominal CT are indicated^(18)^.

### Treatment of GTN

The treatment of GTN essentially consists of chemotherapy, for which the histopathological diagnosis is not a prerequisite^(1,19)^. However, the treatment is preceded by anatomical staging (Table 1), which allows the results to be compared among various referral centers^(20)^, as well as allowing the determination of the FIGO risk score for chemoresistance^(7)^, as shown in Table 2, which is fundamental to choosing the treatment strategy, except in cases of PSTT or ETT^(21)^.

**Table 1:** 2000 FIGO staging system for GTN.

Stage I	Disease confined to the uterus
Stage II	GTN has spread outside the uterus but limited to genital system
Stage III	GTN has spread to the lungs, with or without involvement of the genitourinary system
Stage IV	All other metastatic sites

**Table 2:** 2000 FIGO risk scoring system.

	FIGO risk score			
	0	1	2	4
Age (years)	< 40	≥ 40	-	-
Previous pregnancies	Mole	Abortion	Term	-
Range of pregnancy (months)	< 4	4–6	7–12	> 12
β-hCG pretreatment (mIU/mL)	< 10^3^	10^3^–10^4^	10^4^–10^5^	> 10^5^
Greatest extent of the tumor, including the uterus (cm)	< 3	3–5	≥ 5	-
Site of metastasis	Lung	Spleen, kidney	Gastrointestinal tract	Liver, brain
Number of metastasis	0	1–4	5–8	> 8
Previous chemotherapy	-	-	One drug	Two or more drugs

Metastases are identified in approximately 10-19% of patients with GTN, metastases to the lung accounting for 76-87%), followed by those to the vagina (30%), liver (10%), brain (10%), and, to a lesser degree, the kidney, gastrointestinal system, and spleen^(2)^. Most GTN metastases are hematogenous, except for those to the vagina, which occur by contiguous dissemination^(22)^.

For low-risk GTN (FIGO score ≤ 6), the treatment of choice is single-agent chemotherapy. The first-line drugs are methotrexate and actinomycin-D, both of which have been shown to induce remission in 50-90% of cases^(7)^. Patients with high-risk GTN (FIGO score ≥ 7) require treatment with multiple antineoplastic agents, as do those with stage IV disease. One of the most common treatment regimens is the combination of etoposide, methotrexate, and actinomycin-D, alternating weekly with cyclophosphamide plus vincristine^(23)^. Even patients with metastatic disease have a good prognosis, with cure rates greater than 90%, especially if treated at a referral center^(4)^.

Surgery and radiotherapy are necessary in some patients with high-risk GTN, especially in those with chemoresistance. Because PSTT and ETT respond poorly to chemotherapy, they should be treated with chemotherapy and hysterectomy, sometimes including pelvic lymphadenectomy^(24)^. In cases of PSTT and ETT, the five-year survival rate ranges from 100%, for patients with localized disease, to 50- 60%, for those with metastatic disease^(4)^.

The rate of recurrence of GTN is approximately 3%, and such recurrence is most common during the first year of follow-up. Therefore, careful monitoring of hCG and contraception are essential. Braga et al. reported that there is increased risk of miscarriage and adverse perinatal outcome during the first 6 months after the end of chemotherapy, stating that affected patients should avoid becoming pregnant for 6 months after the final chemotherapy session^(25)^. Thereafter, fertilization is apparently unaffected, successful pregnancies having been reported in more than 80% of patients undergoing chemotherapy for the treatment of GTN^(26)^.

## RADIOLOGICAL CHARACTERISTICS OF GTD

### Ultrasound in molar pregnancy

Ultrasound is the initial imaging test employed in the investigation of cases of molar pregnancy. A serum β-hCG level that is abnormally high for early gestational age raises suspicion of hydatidiform mole. In such cases, ultrasound is mandatory in order to exclude this form of reproductive counterfeiting^(27)^.

**Figure 1:**
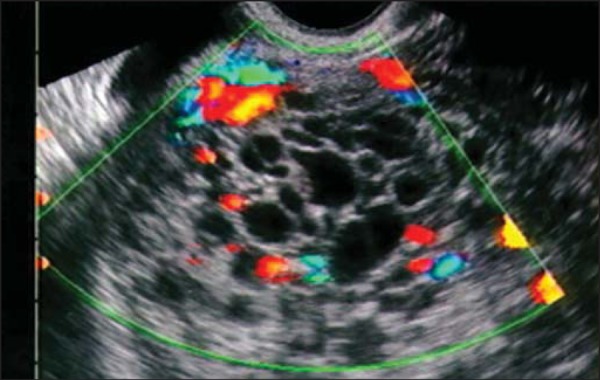
Transvaginal ultrasound in a patient with bleeding at 14 weeks of pregnancy, showing an enlarged uterus with an endometrial cavity filled with amorphous material with multiple anechoic areas, suggestive of complete hydatidiform mole. Note the absence of embryonic tissue and its attachments. Note that, in the Doppler flow study, there was no vascular flow among the vesicles, indicating their avascular nature.

The ultrasound examination can be transabdominal or transvaginal. Due to its higher spatial resolution and anatomical proximity to the study area, transvaginal ultrasound provides a detailed study of uterine lesions, including the morphology and degree of invasion^(2)^. Ultrasound of hydatidiform mole can reveal an intrauterine mass of variable echogenicity, although most hydatidiform moles are echogenic^(1,27,28)^, with multiple, small, diffusely distributed vesicles within an enlarged uterus^(1,2,19)^. These classic vesicular lesions, the aspect of which has been described as "snow storm", "bunch of grapes", or "granular", range from 1 mm to 30 mm in size and represent the hyperplastic and hydropic villi seen on transvaginal ultrasound during the first trimester (Figure 1). In the second trimester, the anechoic spaces increase in number and size, thus facilitating the diagnosis, including that made by transabdominal ultrasound^(27)^. At some facilities, despite the superiority of transvaginal imaging, pre-chemotherapy molar pregnancy patients often do not undergo transvaginal ultrasound due to the chance that a vaginal metastasis, which has a risk of major bleeding^(2)^, will be encountered.

On ultrasound imaging, it can be difficult to differentiate between complete and partial hydatidiform moles. The sensitivity of ultrasound is higher for the detection of complete hydatidiform mole and increases after 16 weeks of pregnancy^(7)^. The diagnosis should always be confirmed by histopathological examination of tissue obtained through uterine evacuation^(2)^. Sebire et al.^(29)^ reviewed 155 histopathological examinations of tissue obtained from patients with ultrasound suspicion of molar pregnancy. Only 34% of those had a confirmed diagnosis, the vast majority being defined as abortion. The authors found the positive predictive value of ultrasound to be higher in cases of complete hydatidiform mole than in those of partial hydatidiform mole (58% vs. 17%).

In a study involving the largest sample of patients with suspected hydatidiform mole ever studied (> 1000 patients), the role of ultrasound in the diagnosis of molar pregnancy was evaluated^(30)^. The authors reported that the sensitivity, specificity, positive predictive value, and negative predictive value of ultrasound in identifying molar pregnancy were 44%, 74%, 88%, and 23%, respectively.

**Figure 2:**
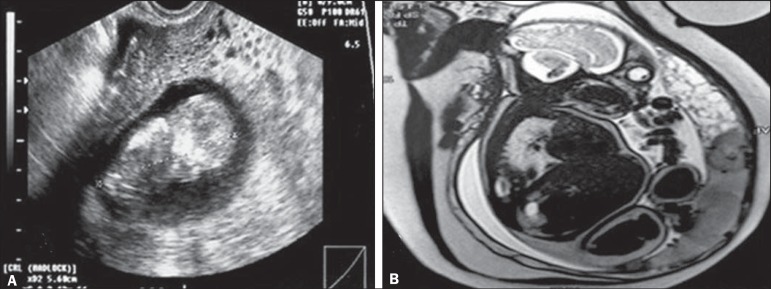
**A:** Routine transvaginal ultrasound at 12 weeks of pregnancy, showing a fetus with normal morphology and a placental area suggestive of complete hydatidiform mole. **B:** MRI scan at 27 weeks of pregnancy, showing a fetus without morphological anomalies and two distinct placental areas: one with a normal appearance; and the other characterized by multiple, hyperintense vesicular areas, suggesting a twin molar pregnancy. Because of severe preeclampsia-severe hypertension; hemolysis/elevated liver enzymes/low platelet count syndrome; and acute pulmonary edema—a cesarean section was performed at 28 weeks of pregnancy. The extremely premature neonate survived without sequelae. After the cesarean section, the patient showed a satisfactory evolution and was discharged from the post-molar pregnancy follow-up after 12 months of treatment with normal-dose â-hCG, without chemotherapy.

In cases of complete hydatidiform mole, there is no fetus or fetal material, except in those rare cases (1-2%) in which there is a dizygotic twin pregnancy with a diploid pattern (Figure 2). In such cases, partial hydatidiform mole with trisomy is differentiated by identifying a separate, normal, placenta^(1,2,27)^.

**Figure 3:**
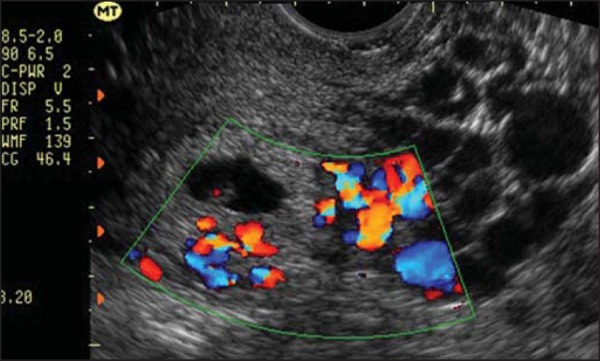
Transvaginal ultrasound showing an embryo and a deciduous area filled with anechoic images suggestive of partial hydatidiform mole. Because the patient was clinically stable and there was a fetal heartbeat, we opted for watchful waiting, until fetal death was confirmed at 14 weeks of pregnancy, indicating the induction of a molar abortion.

Partial hydatidiform mole presents as thickened placental tissue containing various anechoic cystic lesions^(31)^, and some cases can present amniotic membranes and a functional umbilical circulation, as depicted in Figure 3^(32)^. It is usually accompanied by malformation of the gestational sac or of the fetus, which can have characteristics such as hydrocephalus, syndactyly, cleft lip, and growth restriction^(12)^. Hydropic degeneration of the placenta, which occurs in some cases of abortion, produces images of the placenta similar to those seen in cases of partial hydatidiform mole, thus increasing the difficulty of making the diagnosis with ultrasound^(3)^.

**Figure 4:**
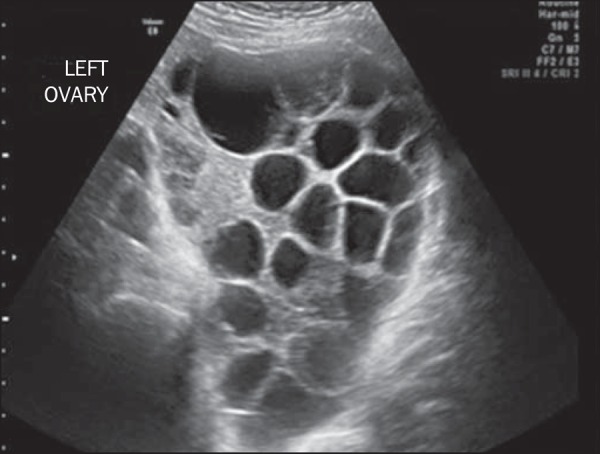
Pelvic ultrasound showing a massive theca lutein cyst in a patient with complete hydatidiform mole.

In more than 40% of cases, theca lutein cysts over 6 cm in diameter can be visualized. Characteristically, they are bilateral and multilocular (Figure 4); they typically do not require treatment^(1)^. Theca lutein cysts, which derive from ovarian hyperstimulation caused by high circulating levels of gonadotropins, generally regress after a few months, in parallel with normalization of β-hCG levels. In rare cases, there is adnexal torsion with acute vascular abdomen or rupture that results in hemoperitoneum, both of which call for immediate treatment^(24)^.

**Figure 5:**
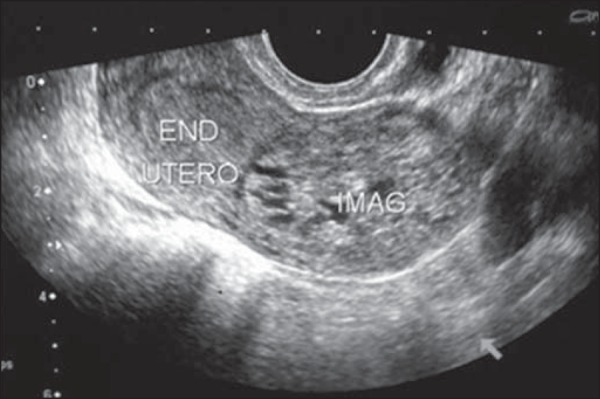
Transvaginal ultrasound showing an empty endometrial cavity, adjacent to a large quantity of amorphous, anechoic, multivesicular material, suggesting tubal molar pregnancy, which was subsequently confirmed by histopathology.

Although quite rare, tubal molar pregnancy, as depicted in Figure 5, does occur^(15)^. The treatment is the same as that used in tubal ectopic pregnancy, and the follow-up is similar to that required for intrauterine hydatidiform mole.

Some cases of mole show nonspecific alterations on Doppler flow studies, although ultrasound is more widely used in the evaluation of cases of GTN^(2,19)^.

### Ultrasound in GTN

**Figure 6:**
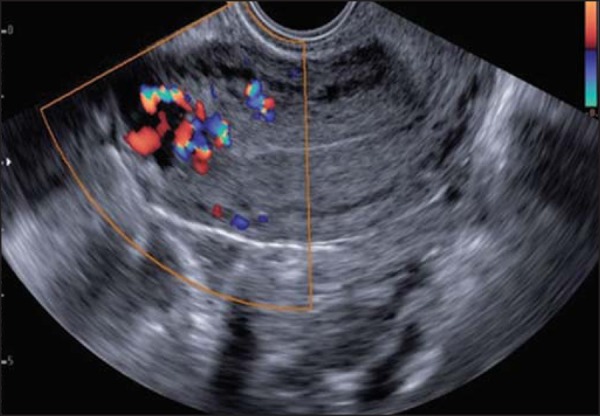
Transvaginal ultrasound, acquired during post-molar pregnancy followup, when hCG levels were elevated. Note the presence of hypoechoic areas in the myometrium, resembling the hypervascular “Swiss cheese” aspect, suggestive of an invasive mole, on the Doppler flow study.

Myometrial invasion is best defined by transvaginal ultrasound. Invasive moles, choriocarcinoma, and PSTT have a similar appearance-a focal myometrial mass that can be either uniformly echogenic or hypoechoic (Figure 6), as well as being complex or multicystic^(33)^. Anechoic spaces within the mass are related to hemorrhage, tissue necrosis, cysts, or vascular spaces^(2,19)^. Patients with more advanced disease can present with an enlarged uterus, with lobulated, heterogeneous contours, or a pelvic mass that extends to adjacent organs^(28)^. The volume of the uterine lesion must be determined because it has an established relationship with the size of the tumor and the risk of chemoresistance^(34)^.

The changes seen on ultrasound of GTN are nonspecific, and the differential diagnosis should include other pelvic malignancies, as well as myoma and adenomyosis^(33)^. Correlating clinical history with β-hCG levels and with Doppler flow study findings is essential to making the correct diagnosis^(28)^.

Because GTN subtypes are indistinguishable from each other in imaging studies, the diagnostic hypothesis follows a specific sequence. The initial assumption is invasive mole. However, if metastasis is detected, the focus shifts to choriocarcinoma. Chemotherapy is started even before histological confirmation has been obtained, and both entities are treated with the same chemotherapy regimen^(1)^. Effective post-treatment ultrasound usually identifies a hypoechoic lesion that progressively decreases in size^(28)^.

A diagnosis of PSTT is strongly suggested when there are changes that are characteristic of GTN in combination with low levels of β-hCG. Unlike invasive mole and choriocarcinoma, PSTT is distinguished by its relative chemoresistance and the potential need for surgical treatment^(2,27)^.

### Doppler flow studies in GTN

Color and spectral Doppler flow studies are used together with an ultrasound gray scale in the assessment of GTN and in its post-treatment follow-up^(35)^. The vasculature has a chaotic appearance, with color distortion and vascular changes, due to arteriovenous communications and neovascularization of the myometrial mass^(19)^. The uterine vessels can be evaluated by determining their wave patterns, peak systolic velocity, resistance index (RI), and pulsatility index (PI).

In the evaluation of uterine arteries during the first trimester of a normal pregnancy, Doppler flow studies show high impedance wave patterns with low diastolic velocities, except at the placental implantation site. Because of physiological vascular invasion by trophoblastic tissue, the placental implantation site has a low-impedance flow^(2,27)^. In the second and third trimesters, there is reduced impedance due to the physiological advance of arterial invasion of the trophoblast. However, in the first trimester of a molar pregnancy, there is high flow velocity and low-impedance wave patterns due to the greater arterial invasion caused by abnormal proliferation of the trophoblast^(27,28)^. Zhou et al.^(31)^ compared the RIs of the uterine arteries in patients with hydatidiform mole (complete or partial) with those of the uterine arteries in patients with GTN, finding that the RIs were lower in the latter group. Although there is no consensus on the values, an RI < 0.4 and a PI < 1.5 are thought to be indicative of a uterine artery with low resistance, which is typical of GTN^(31)^.

It should be borne in mind that the color Doppler ultrasound features of GTN are nonspecific. Other conditions can have a similar appearance, such conditions including the presence of residual trophoblastic tissue from a miscarriage or ectopic pregnancy, pelvic inflammatory disease, other uterine malignancies, diverticulitis or appendicitis with uterine abscesses, and uterine arteriovenous malformations^(19)^.

The PI of the uterine artery is an indirect measure of functional vasculature of the tumor, being considered a predictor of resistance to chemotherapy, especially to methotrexate, regardless of the FIGO score^(35)^. It is known that a low PI indicates a higher number of arteriovenous communications and greater neovascularization. Sita-Lumsden et al.^(36)^ showed that patients with a PI ≤ 1 for the uterine artery have an absolute risk of methotrexate resistance of 67%, compared with 42% for those with a uterine artery PI > 1.

Doppler flow studies can also be used to evaluate the response to chemotherapy. *Pari passu* to the drop in the serum levels of β-hCG, regression of the vascular cystic spaces of the intramyometrial mass is also seen. During the post treatment follow-up, ultrasound can also serve to diagnose disease complications such as uterine arteriovenous malformations^(2,27)^.

### Chest X-ray in GTN

**Figure 7:**
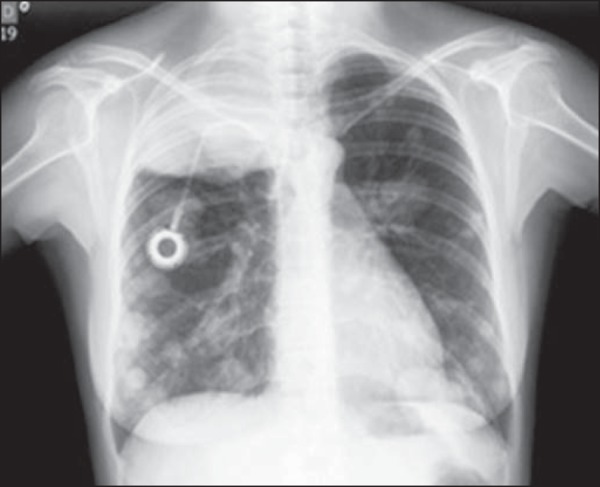
Posteroanterior chest X-ray, acquired during follow-up, showing numerous, dense, bilateral metastatic nodules, of varying sizes.

Chest X-ray is the examination of choice for the initial evaluation of metastatic lung cancer. There are three basic forms of radiological presentation of metastatic pulmonary GTN: typical, alveolar, and embolic. The typical image is that of dense nodules with well-defined contours, usually multiple and bilateral (Figure 7). When there is cellular involvement, a chest X-ray can show multiple nodules and small, poorly defined opacities, similar to the images produced by inflammatory processes. Radiographic images showing pulmonary hypertension and cardiovascular changes suggest the occurrence of thromboembolic phenomena^(37)^. Other, rarer, radiographic changes have also been associated with GTN, including pleural effusion, interlobular septal thickening, cavitations, and air bronchogram^(38)^.

Even after effective chemotherapy treatment, lung nodules can still be seen on chest X-rays. If there is no growth of the tumor mass during the radiological follow-up and β-hCG levels remain in the normal range, such nodules should be considered residual, probably related to tissue necrosis and not to active neoplasia^(39)^.

### CT in GTN

In cases of GTN, the use of CT is fundamental for the investigation of sites of metastasis, except for those in the vagina or the brain^(2)^. It is noteworthy that the lung is the most common site of GTN^(40)^ and that choriocarcinoma is the GTN subtype most often identified^(41)^.

On CT, GTN confined to the uterus can be described as a low-attenuation lesion within an enlarged uterus^(42)^. Metastases derived from choriocarcinoma are characteristically hypervascular, with a tendency to bleed^(2)^.

**Figure 8:**
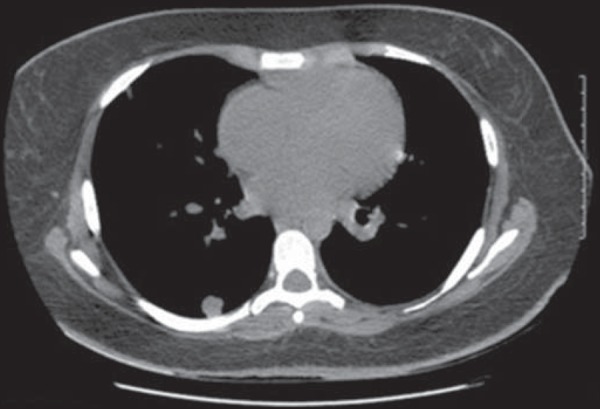
Contrast-enhanced CT of the chest, acquired during follow-up, showing numerous metastatic lung lesions. Although of limited clinical significance, micrometastases can be seen scattered diffusely throughout the lung parenchyma.

The pulmonary lesions of GTN seen on CT are typically rounded and larger than 3 cm in diameter. Such lesions rarely form cavities (Figure 8). They can be single lesions (otherwise usually found in numbers of less than 10), and they have a miliary aspect^(40)^. Pleural, endovascular, and endobronchial lesions have also been described^(2)^.

Despite the increased sensitivity of CT in detecting micrometastases of GTN, the FIGO recommends chest Xray as the initial test for pulmonary evaluation. Studies have shown that nodules can persist after effective chemotherapy, without affecting the prognosis^(39)^.

**Figure 9:**
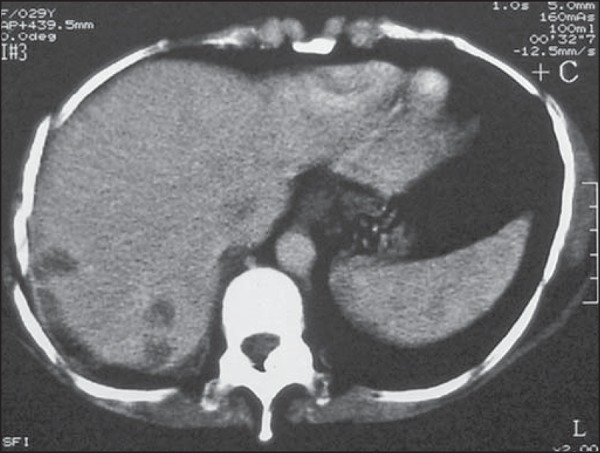
CT of the abdomen showing three hypointense, hypovascular lesions with peripheral enhancement in a patient with GTN. The patient evolved to liver rupture, hemoperitoneum, and death. The autopsy revealed metastatic choriocarcinoma of the liver.

In patients classified as high risk and showing metastasis to the lung or vagina, abdominal CT is recommended^(20)^. If there is liver involvement, the lesions are usually multiple, heterogeneous, and hypointense, with a high avidity for intravenous contrast in the arterial phase (Figure 9), and hemorrhagic transformation is common. These metastatic liver lesions are not easily distinguished from other hypervascular liver tumors. In order to make that distinction, it should be borne in mind that these neoplasms develop a hypervascular mass with aneurysmal dilatation in the peripheral hepatic arteries, which are best visualized in the arterial phase, whereas persistent vascular lakes are observed in the venous phase. These lesions appear late in the course of the disease and are related to poor prognosis. Although biopsy is contraindicated because of the risk of fatal bleeding^(43)^, these lesions can respond to selective chemoembolization.

Other sites are reported to be affected, including the spleen, kidneys, gastrointestinal tract, and skin. In cases of PSTT, lymph node involvement is common^(4,44)^.

### Positron emission tomography in GTN

**Figure 10:**
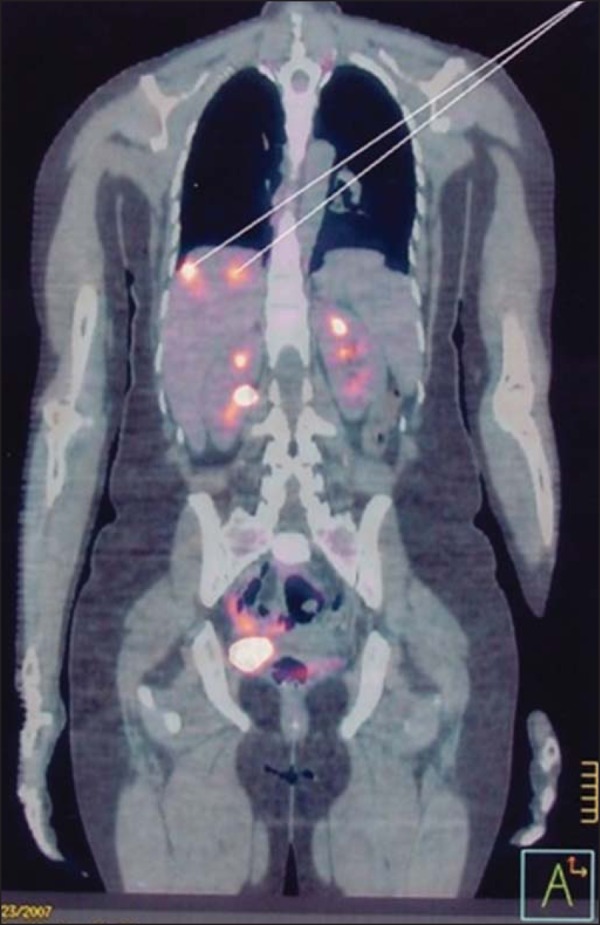
PET scan, using ^18^F-fluorodeoxyglucose, showing intense metabolic tumor activity in the liver, featuring metastatic choriocarcinoma nodules, in a patient under follow-up treatment.

In patients with GTN, studies involving positron emission tomography coupled with CT (PET/CT), using ^18^Ffluorodeoxyglucose, have shown potential not only to determine the extent of the tumor and to identify metastases but also to evaluate the response of high-risk tumors to treatment (Figure 10). The detection of metabolically active disease can reveal occult injuries, confirm a complete response to treatment, and allow GTN recurrence to be evaluated^(44)^. In a study conducted in the United Kingdom, nine patients underwent diagnostic PET/CT during the staging of recurrent GTN. The PET/CT helped locate active disease sites in six of those patients, and one patient showed no abnormalities on ultrasound, MRI, or CT^(2)^.

### MRI in GTN

The routine evaluation of GTN does not include MRI, which is reserved for use in complicated and inconclusive cases, such as suspected PSTT, advanced disease, and recurrent disease^(45)^. Through the use of MRI, the location, vasculature, and extent of the tumor can be evaluated with greater accuracy. However, the imaging findings are nonspecific and can be difficult to distinguish from those of, for example, retained products of conception or an ectopic pregnancy with GTN^(46)^.

An MRI scan of a hydatidiform mole obtained during the first trimester shows little or no abnormality. Such abnormalities are best viewed in the second trimester. Contrast-enhanced T1-weighted images can show a mass with a signal equal to or slightly more intense than that of the adjacent myometrium, containing small, distributed diffusely cystic spaces, reflecting the vesicular nature of the tumor^(46)^. The presence of foci with hyperintense signals is probably due to hemorrhagic foci within the lesion. On T2-weighted images, the tumor presents a heterogeneous, hyperintense mass, with a "bunch of grapes" appearance, that distends the uterus and endometrial cavity^(47)^.

Myometrial invasion can be suspected when the lesion crosses the myoendometrial border and the transitional zone becomes undefined. These changes have also been identified in routine cases of miscarriage and in patients who have recently undergone curettage. Some studies have shown a direct correlation between such architectural deregulation and circulating levels of β-hCG, levels > 1500 mIU/mL being accompanied by a greater change in uterine architecture and a greater tumor burden, whereas levels < 500 mIU/mL are usually accompanied by no changes on MRI scans^(46)^.

**Figure 11:**
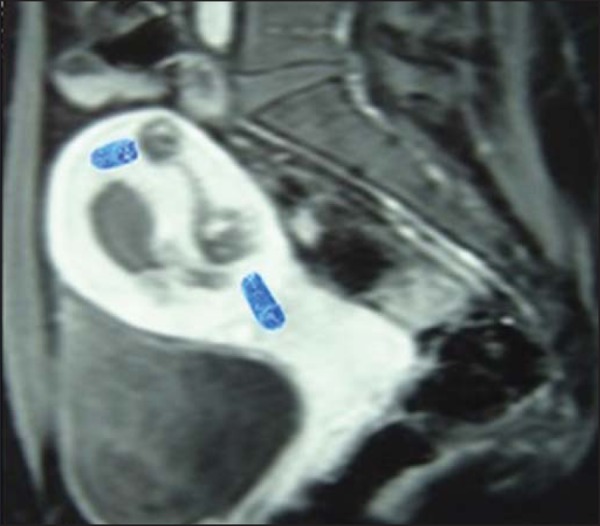
Gadolinium contrast-enhanced MRI scan of the pelvis, showing, hypointense lesions with avid uptake and vascular dilation in the myometrium, sometimes in close contact with the uterine effusion, in patients treated for GTN.

Due to the high degree of vascularization, T1-weighted and T2-weighted images both show various spaces with tortuous flows, consistent with vessels that pass through the tumor mass, myometrium, parametrium, or attachments, as well as with engorgement of the iliac vessels^(48)^. The hemorrhagic foci usually have a high signal intensity on T1-weighted images and can best be distinguished from active disease by dynamic contrast-enhanced MRI, as shown in Figure 11^(47)^.

Chemotherapy reduces the volume and vascularization of the tumor, and MRI scans usually show normal images by 6-9 months after the end of treatment^(48)^. Vascular malformations can be detected years after treatment^(2)^.

On MRI scans, PSTT can present in a hypervascular form or a hypovascular form. In the hypervascular form, the tumor has an isointense signal on T1-weighted images, a slightly hyperintense signal on T2-weighted images, and avid uptake after the administration of contrast (gadolinium). Many vessels are visualized in all MRI scans. In the hypovascular form, the tumor has less volume, as well as a higher signal intensity on T1- and T2-weighted images, and a lower rate of uptake of contrast. There is no evident vascularization^(49)^.

In the metastatic evaluation of GTN, MRI also plays a role. It is superior to ultrasound in identifying parametrial and vaginal invasion^(2)^. On T2-weighted images, a hyperintense mass can be seen in the parametrial tissue, whereas vaginal involvement presents as a bulging into the fornix with a hyperintense signal and ill-defined borders^(48)^.

**Figure 12:**
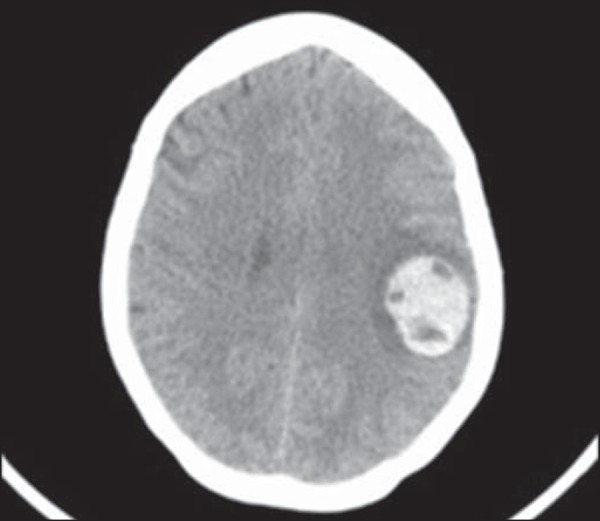
MRI scan of the brain, showing a mass suggestive of metastatic choriocarcinoma, in a 32 year-old patient presenting with headache, speech articulation disorder, and dysphagia.

Patients with pulmonary metastases of GTN are also submitted to evaluation of the brain tissue. In the brain, there are typically multiple lesions, primarily located in the parietal lobe at the junction between the white and gray matter. The images seen on an MRI scan have varying characteristics (Figure 12), depending on the duration of the associated bleeding^(20,50)^. The image is improved by contrast administration^(2,20,50)^.

### Angiography in GTN

Conventional angiography can be used for the embolization of vaginal and liver metastases. Another use of conventional angiography is in the management of cases of uterine arteriovenous malformations after GTN in patients who are symptomatic and wish to conceive, given that selective embolization of such malformations, via the uterine artery, has provided auspicious results. Traditionally, such patients have undergone hysterectomy and ligation of the uterine arteries, making subsequent reproduction impossible^(2,27)^.

## CONCLUSION

Albeit a relatively uncommon disease with malignant potential, GTD is almost always curable. Although the β-hCG level is an excellent biomarker, it cannot be used in isolation to make the diagnosis of GTD. Ultrasound is the firstline examination in the diagnosis of molar pregnancy. When combined with Doppler flow studies, it is useful not only in the evaluation of GTN but also in the evaluation of the response to treatment and in the detection of GTN recurrence. Screening for metastatic GTN should include chest X-ray and CT. In complicated cases, MRI is used as an ancillary method to assess the extent of the tumor. To date, there have been few studies involving the use of PET/CT in cases of GTN. However, PET/CT has proven efficient in identifying occult neoplasia. It is evident that the radiologist plays a fundamental role throughout the course of the treatment of patients with GTD, from diagnosis to follow-up after cure.
